# Brief exposure to obesogenic diet disrupts brain dopamine networks

**DOI:** 10.1371/journal.pone.0191299

**Published:** 2018-04-26

**Authors:** Robert L. Barry, Nellie E. Byun, Jason M. Williams, Michael A. Siuta, Mohammed N. Tantawy, Nicole K. Speed, Christine Saunders, Aurelio Galli, Kevin D. Niswender, Malcolm J. Avison

**Affiliations:** 1 Vanderbilt University Institute of Imaging Science, Vanderbilt University Medical Center, Nashville, Tennessee, United States of America; 2 Department of Radiology and Radiological Sciences, Vanderbilt University Medical Center, Nashville, Tennessee, United States of America; 3 Department of Pharmacology, Vanderbilt University Medical Center, Nashville, Tennessee, United States of America; 4 Department of Medicine, Vanderbilt University Medical Center, Nashville, Tennessee, United States of America; 5 Department of Molecular Physiology and Biophysics, Vanderbilt University Medical Center, Nashville, Tennessee, United States of America; 6 Vanderbilt Brain Institute, Vanderbilt University Medical Center, Nashville, Tennessee, United States of America; 7 Department of Psychiatry, Vanderbilt University Medical Center, Nashville, Tennessee, United States of America; 8 Tennessee Valley Healthcare System, Nashville, Tennessee, United States of America; University of Leicester, UNITED KINGDOM

## Abstract

**Objective:**

We have previously demonstrated that insulin signaling, through the downstream signaling kinase Akt, is a potent modulator of dopamine transporter (DAT) activity, which fine-tunes dopamine (DA) signaling at the synapse. This suggests a mechanism by which impaired neuronal insulin receptor signaling, a hallmark of diet-induced obesity, may contribute to impaired DA transmission. We tested whether a short-term (two-week) obesogenic high-fat (HF) diet could reduce striatal Akt activity, a marker of central insulin, receptor signaling and blunt striatal and dopaminergic network responsiveness to amphetamine (AMPH).

**Methods:**

We examined the effects of a two-week HF diet on striatal DAT activity in rats, using AMPH as a probe in a functional magnetic resonance imaging (fMRI) assay, and mapped the disruption in AMPH-evoked functional connectivity between key dopaminergic targets and their projection areas using correlation and permutation analyses. We used phosphorylation of the Akt substrate GSK3α in striatal extracts as a measure of insulin receptor signaling. Finally, we confirmed the impact of HF diet on striatal DA D2 receptor (D2R) availability using [^18^F]fallypride positron emission tomography (PET).

**Results:**

We found that rats fed a HF diet for only two weeks have reductions in striatal Akt activity, a marker of decreased striatal insulin receptor signaling and blunted striatal responsiveness to AMPH. HF feeding also reduced interactions between elements of the mesolimbic (nucleus accumbens–anterior cingulate) and sensorimotor circuits (caudate/putamen–thalamus–sensorimotor cortex) implicated in hedonic feeding. D2R availability was reduced in HF-fed animals.

**Conclusion:**

These studies support the hypothesis that central insulin signaling and dopaminergic neurotransmission are already altered after short-term HF feeding. Because AMPH induces DA efflux and brain activation, in large part via DAT, these findings suggest that blunted central nervous system insulin receptor signaling through a HF diet can impair DA homeostasis, thereby disrupting cognitive and reward circuitry involved in the regulation of hedonic feeding.

## Introduction

Brain dopamine (DA) signaling plays a key role in the regulation of complex behaviors including feeding. Similarities in patterns of DA dysfunction in obese individuals and those with substance use disorders revealed by neuroimaging [1,2] and preclinical genetic and pharmacological manipulations [3,4] strongly suggest that altered DA signaling may predispose to, promote, and/or maintain obesogenic feeding. Changes in DA D2 receptor (D2R) expression levels and signaling in obesity have been well documented [1,2,4]. However, in addition to DA neurotransmission through DARs, central insulin signaling in DA and norepinephrine (NE) terminals plays an important role in regulating DA (and NE) tone by tuning the levels of DA transporter (DAT) and NE transporter (NET) membrane expression and activity [5–9]. The frequent comorbidity of impaired insulin signaling with obesity suggests a mechanism whereby an obesogenic/diabetogenic diet, by blunting central insulin signaling, disrupts brain networks whose integrity requires optimal monoaminergic tone.

We have shown previously that amphetamine (AMPH)-evoked striatal DA release measured by high-speed chronoamperometry (HSCA) is a reporter of striatal DAT activity *in vivo*, and that, consonant with decreased DAT surface expression and activity, the functional magnetic resonance imaging (fMRI) response to AMPH is blunted with insulin depletion and subsequent reduction of downstream Akt (protein kinase B) signaling in a rat model of Type 1 diabetes mellitus [5]. Next, we determined the molecular relationship between insulin receptor signaling and striatal DAT surface expression and function in a diet-induced obesity (DIO) model. A 4-week high fat (HF) diet impaired striatal and nigral Akt signaling, indicative of blunted insulin receptor signaling, and significantly reduced DAT surface expression and HSCA-measured striatal DA clearance in the HF-fed obese rats [8]. Rescue of nigral Akt activity in HF animals by viral overexpression of insulin receptor substrate 2 (IRS2) restored striatal DAT surface expression and DA clearance, and reduced hyperphagia, while pharmacological inhibition of Akt in normal rats blunted DAT activity, supporting a causal link between impaired insulin signaling and striatal DAT activity [8]. We then reasoned that the onset of striatal insulin receptor signaling changes could be induced by a brief HF diet, and that this diet could lead to an early disruption of DAT expression and DA neurotransmission in striatal and extrastriatal areas. Specifically, we hypothesized that (1) diet-induced decreases in neuronal insulin receptor signaling would impair DA clearance and disrupt DA homeostasis in key brain areas mediating reward (nucleus accumbens, NAc) and motivated behavior (caudate putamen, CP); and (2) that these impairments would consequently disrupt functional connections between these brain areas and their targets. To test these hypotheses, we examined the effects of a two-week HF diet in rats on striatal DAT activity, using AMPH as a probe in a fMRI assay that allowed us to map the disruption in AMPH-evoked functional connectivity between key dopaminergic targets and their projection areas using correlation and permutation analyses. We also tested the impact of a short-term HF diet on striatal DA D2R availability using [^18^F]fallypride positron emission tomography (PET).

## Materials and methods

### Ethics statement

All procedures were approved by the Vanderbilt University Medical Center Institutional Animal Care and Use Committee and conducted in accordance to the National Institutes of Health Guide for the Care and Use of Laboratory Animals.

### Animals and diet

Individually housed male Sprague-Dawley rats (275–300 g; Harlan, Indianapolis, IN) in solid-bottomed cages lined with corncob bedding were acclimated to a low-fat (LF) control diet for 7 days (10% fat; D12450B, Research Diets, New Brunswick, NJ). Half of the rats were then switched to a micronutrient-matched HF diet (60% fat; D12492, Research Diets) for an additional 14 days. Food intake and body weights were measured daily. Caloric consumption was calculated using the conversion factors of 5.24 kcal/g for HF diet and 3.85 kcal/g for the LF diet. Body composition, gauged by MR spectroscopy (Echo Medical Systems, Houston, TX), was determined weekly.

### Striatal Akt activity assay

Rats were decapitated without anesthesia, and striatal synaptosomes were prepared and assayed as previously described [6] using an *in vitro* Akt kinase function assay kit (BioVision, Mountain View, CA) for detecting phosphorylation of its substrate, recombinant glycogen synthase kinase 3 alpha (GSK3α). As the neuronal insulin receptor signals through cytosolic IRS2, which, in turn, activates Akt to phosphorylate GSK3α, levels of phospho-GSK3α reflect a downstream marker of insulin receptor signaling [6]. The Akt kinase assay was performed per the manufacturer's protocol. Protein (∼400 μg) from synaptosome lysates was immunoprecipitated with anti-Akt monoclonal antibody from the Akt assay kit. Activity of the immunoprecipitated kinase was measured *in vitro* using exogenous recombinant GSK3α that was added as the substrate. Levels of phosphorylated GSK3α were determined by immunoblotting using phospho-specific antibodies to GSK3α (Ser-21).

### Functional MRI

Anesthetized (2–2.5% isoflurane), tracheotomized rats were implanted with intraperitoneal (ip) and bilateral femoral intravenous (iv) catheters, then mechanically ventilated (30% O_2_:70% N_2_O) under continuous isoflurane, placed in an MR-compatible stereotaxic holder, and paralyzed (pancuronium bromide 2 mg/kg, ip; Sigma, St. Louis, MO). They were maintained under 0.88% isoflurane for functional MRI. Respiration, temperature, end-tidal CO_2_, and heart rate were continuously monitored. Studies were performed on a 4.7 Tesla Inova scanner (Agilent, Palo Alto, CA), using a 20-mm radio frequency surface coil transceiver. A high-resolution fast spin echo structural image was acquired for coregistration of functional activation maps [field of view (FOV) = 35 × 35 mm^2^; in-plane resolution = 137 × 137 μm^2^; repetition time (TR) = 2340 ms; echo spacing = 8 ms; echo train length = 8; number of excitations = 2; slice thickness = 2 mm (7 slices)]. A coronal multi-gradient echo R2* mapping sequence was used for functional cerebral blood volume (CBV) mapping [FOV = 35 × 35 mm^2^; in-plane resolution = 273 × 273 μm^2^; TR = 210 ms; echo time (TE) = 3, 8, 13, 18, 23, 28 ms; flip angle = 27°; number of excitations = 2; slice thickness = 2 mm (7 slices); acquisition time = 1 min per volume]. Following collection of a reference transverse relaxation rate R2*_0_ map, iron oxide nanoparticles (MION, 30 nm diameter; BioPal, Worcester, MA; 0.5 ml/kg IV; 7 mg iron/kg, iv) were injected into the right femoral vein and allowed to equilibrate for 5 min. A time series of 40 consecutive R2* maps was then collected, with injection of AMPH (3 mg/kg, iv) or saline (1 ml/kg, iv) via the left femoral catheter after the 10th image.

#### Functional MRI data analysis

R2* images generated from multi-gradient echo data fit to a mono-exponential decay on a voxel-wise basis were used to map the time course of percent CBV change following AMPH or saline using %∆CBV = ∆CBV(t)/CBV_0_ × 100 = (R2*(t)–R2*_post_)/∆R2*_0_ × 100 where R2*_post_ is mean R2* during the baseline period post-MION, pre-saline/AMPH and ∆R2*_0_ is change in R2* following MION injection. Several cortical and subcortical brain regions of interest (ROIs) were selected *a priori* using the Paxinos and Watson rat brain atlas [10] for calculation of mean %ΔCBV time courses. CBV time series data from each ROI were averaged across hemispheres, and mean %ΔCBV for each group over time points 10–30 min were compared using a one-way ANOVA followed by a Newman-Keuls post-hoc test. Group mean %ΔCBV maps were generated after affine warping (3dWarpDrive) of functional maps to a common anatomic reference image using AFNI (http://afni.nimh.nih.gov/afni) [11]. The %∆CBV response slopes (m) between time points 21 and 40 min were calculated for dorsal and ventral striatum, and the slope values for LF versus HF groups were statistically compared using an unpaired one-tailed t-test. Slope variances represent standard error of the mean.

#### Correlation analysis

Two complementary functional correlation methods were used to assess the strength of interregional correlations: (1) area under the curve (AUC; 5–30 min post-AMPH) and (2) time course (TC; temporal cross correlation of time course 0–30 min post-AMPH) of AMPH-evoked brain activation as measures of the integrity of monoaminergic–particularly dopaminergic–networks for LF- and HF-fed animals. This dual analysis approach was employed because AMPH response curves for each ROI display unique characteristics that reflect variations in amplitude (AUC) and/or temporal structure (TC). For this reason, correlation matrices generated by these two approaches are not expected to be identical but rather reflect complementary features (amplitude and temporal dynamics) that are reproducible within groups and significantly different between groups. For both AUC and TC methods, the Pearson linear correlation coefficient, r, was calculated between each ROI pair in the LF and HF groups. These correlation coefficients were then converted to Z-scores using Fisher’s transformation: Z = ln[(1+r) / (1–r)] / [2*(1 / (N–3))^0.5^] where N represents degrees of freedom. The matrix representations of these values were thresholded at a 2-sided 99% confidence interval (|Z| > 2.576). Permutation analyses [12] were then used to construct confidence intervals and identify Z-scores that were significantly different between LF and HF groups (ΔZ, 1-sided 95% confidence interval, *p* < 0.05).

### [^18^F]Fallypride PET

Rats were anesthetized with 1.5% isoflurane for 5–10 min during which time a tail vein catheter was inserted and 15 MBq/0.2 ml of [^18^F]fallypride was injected, followed by a 0.1 ml saline flush via the catheter. Fifty-five min post-[^18^F]fallypride injection, animals were placed in a microPET Focus 220 (Siemens Preclinical Solutions, Knoxville, TN) and a 60-min dynamic acquisition was started. The 60-min dynamic acquisition was divided into six 600-s frames [13]. All datasets were reconstructed using the 2D ordered subsets expectation maximization (OSEM) algorithm into 128 × 128 × 95 slices with a voxel size of 0.095 × 0.095 × 0.08 cm^3^ after correcting for scatter and attenuation. The resulting images were coregistered to an MRI template [14,15] using AMIDE [16]. Anatomical volumetric ROIs were drawn around the striatum and cerebellum and the resulting time-activity curves were corrected for partial volume effects using recovery coefficients derived from a cone-shaped phantom [13].

#### PET data analysis

PET images representing percent injected dose per gram (%ID/g) summed between 0–60 min (the entire scan duration) were analyzed in AMIDE. The distribution volume ratio (DVR’) [11] for each 60-min scan was estimated using the Logan plot [17] tool in version 2.6 of the commercial PMOD software (PMOD Technologies, Zurich, Switzerland, www.pmod.com) where the cerebellum, which has few or no D2Rs, was taken as the reference tissue.

## Results

### High-fat feeding

Diet-induced obesity was induced through a 14-day 60% lard-based HF diet while control animals were fed a micro-nutrient matched 10% low-fat LF diet. Over 14 days, HF-fed (*n* = 10) vs. LF-fed (*n* = 8) rats consumed more calories (1024±20 vs. 853±20 total kcal; F = 37.0, *p* < 0.001), gained more weight (51.4±2.5 vs. 36.6±5.6 g; F = 6.55, *p* < 0.05), and had significantly higher percentage body fat (10.8±0.42 vs. 8.3±0.66%; F = 10.1, *p* < 0.01). Body weights were 385±10 vs. 368±7 g (F = 1.96, p = 0.18). No significant differences between saline- vs. AMPH-challenged groups were observed for any metabolic endpoint.

### High-fat diet blunts striatal insulin signaling

**Fig 1:**
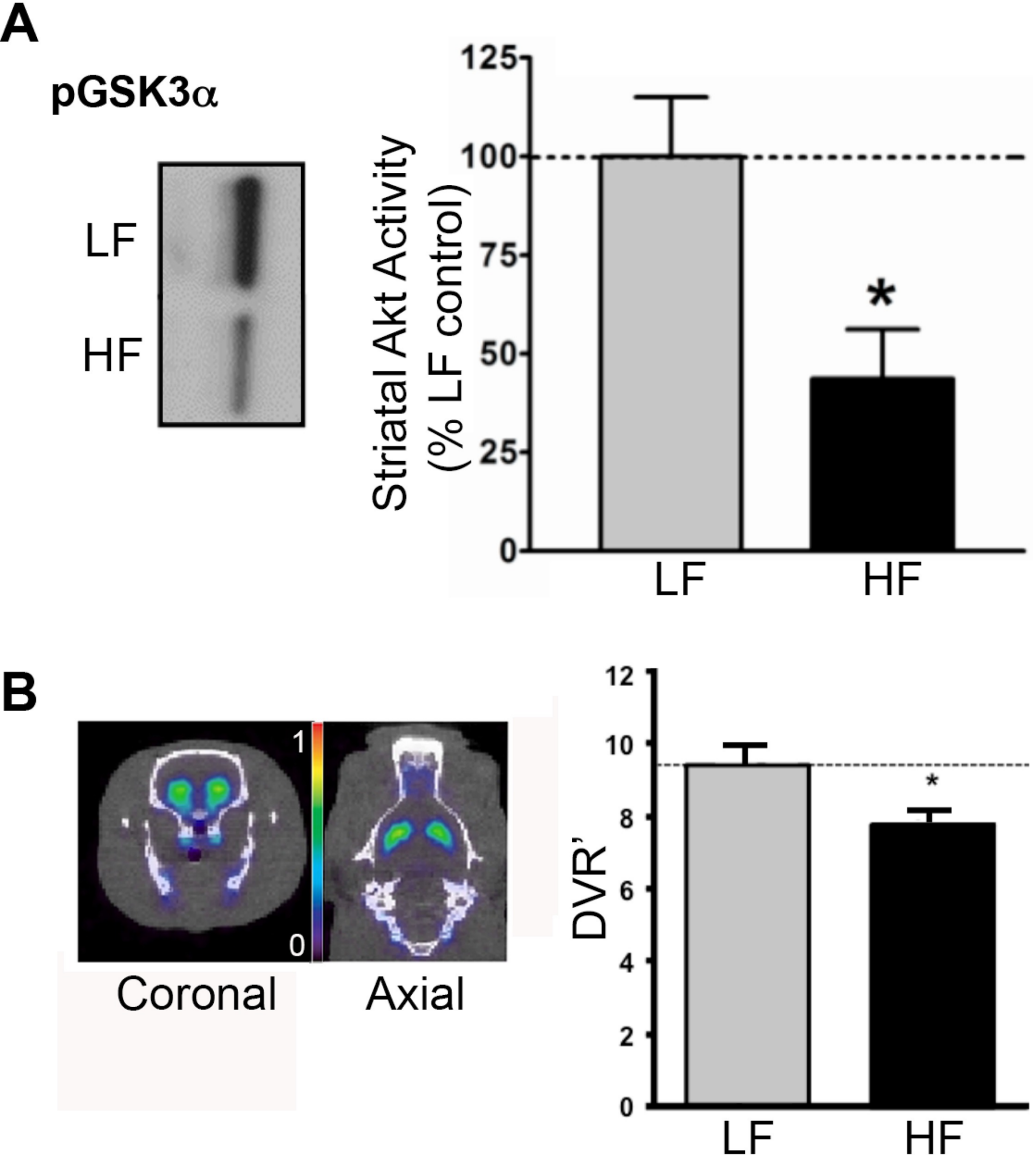
HF diet blunts striatal insulin signaling and D2R availability. (A) Reduction of basal striatal Akt activity in HF- vs. LF-fed animals was determined by phosphorylation of the Akt substrate, exogenous, recombinant GSK3α. Representative phosphoimmunoblots of phospho-GSK3α for HF and LF striatal samples is shown (left panel). The bar graph (right panel) shows data for phospho-GSK3α with each sample normalized to LF control. *p < 0.05, Student’s *t*-test (*n* = 5 animals/group). (B) [^18^F]fallyride PET images (left panel) of coronal and axial views of striatal radiotracer binding in a representative LF animal show green pseudocolor (color bar range indicates 0–1%ID/g). The graph (right panel) represents group-averaged data and shows reduced striatal D2R availability *in vivo* (DVR’—distribution volume ratio) in HF (*n* = 3) vs. LF (*n* = 4) groups, determined by [^18^F]fallyride PET. *p < 0.05, one-tailed Student’s *t*-test (based on *a priori* test of HF < LF).

We and others have previously demonstrated in Type 1 diabetes mellitus and long-term DIO models that insulin receptor signaling through the downstream kinase Akt can regulate surface DAT expression and, thus, DA tone [5–8]. Here we tested whether a 2-week HF diet could decrease striatal insulin signaling. We measured striatal insulin signaling by assaying the ability of Akt, a marker of insulin signaling, to phosphorylate its substrate GSK3α *in vitro* in striatal synaptosomes [6] in a separate cohort. As measured by Western blotting, 14 days of HF diet reduced levels of phosphorylated GSK3α to 44±13% of animals maintained on LF diet (*p* < 0.05), whereas total Akt content did not differ between groups (Fig 1A). These results indicate decreased Akt activity, which is consistent with blunted insulin receptor signaling that can be induced by a relatively short term HF diet.

### High-fat diet decreases striatal dopamine D2 receptor availability *in vivo*

Changes in striatal DA D2R levels and signaling have been well documented in obese humans and rodent DIO models established through a more prolonged high caloric or fat diet [1,2,4]. We compared D2R availability between 14-day HF- vs. LF-fed rats using the high affinity DA D2/3R radioligand for [^18^F]fallypride PET. HF-fed rats had lower striatal [^18^F]fallypride volume of distribution, consistent with reduced D2R availability *in vivo* (*p* < 0.05; Fig 1B). These results, which are consistent with previous findings [1,2,4], indicate that a relatively short-term diet can impact D2R levels.

### High-fat diet attenuates amplitude of striatal and extrastriatal AMPH-evoked brain activation *in vivo*

**Fig 2:**
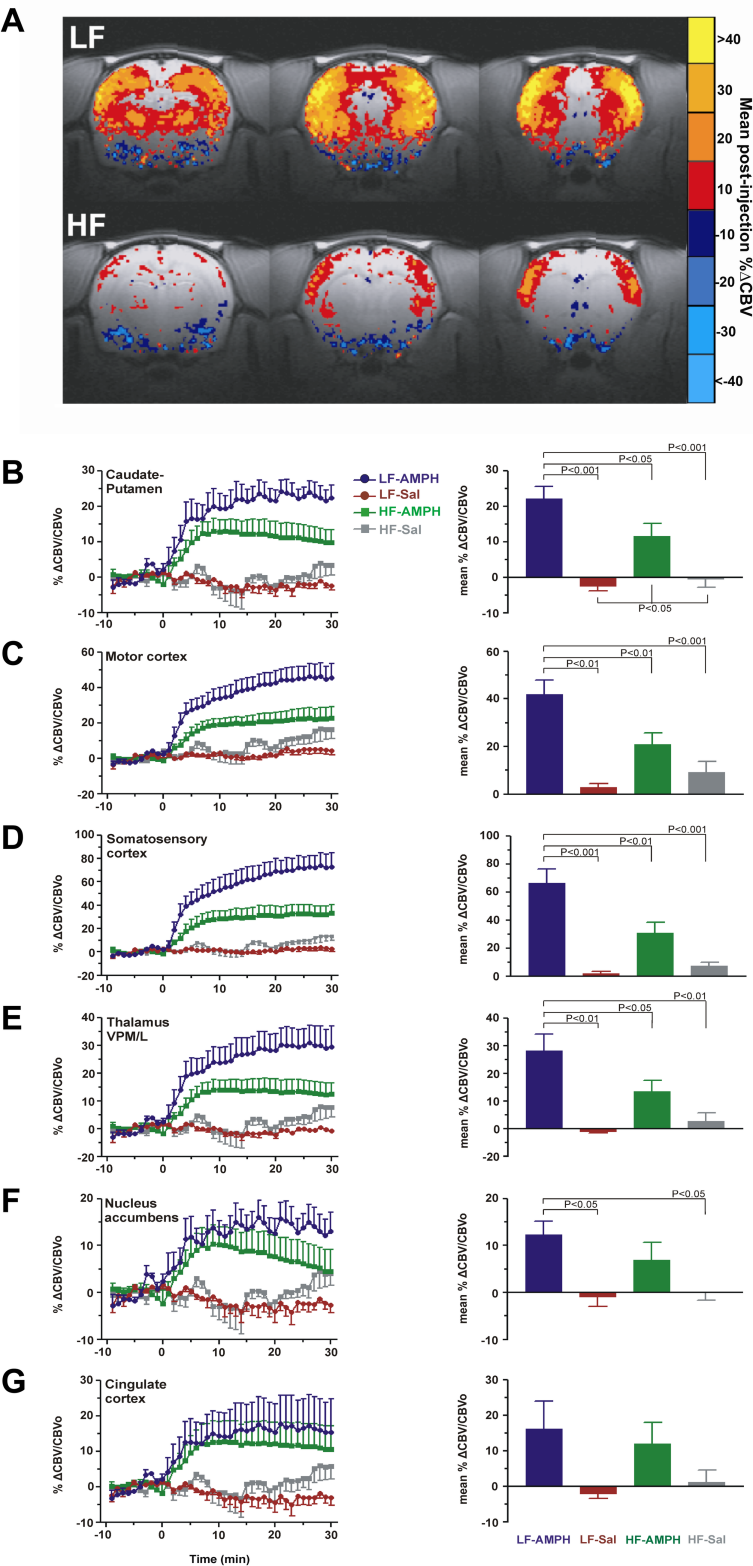
HF diet blunts cortical and subcortical fMRI responses to amphetamine. (A) Blunted amphetamine (AMPH) evoked cerebral blood volume (CBV) changes in HF- vs. LF-fed rats. Group averaged CBV responses for HF- and LF-fed animals shown for three coronal slices centered on caudate putamen (Bregma -2.8, -0.8, +1.2) in both diet groups. Maps depict mean %CBV change over baseline for 15 min post-AMPH. (B-G) Time courses of %∆CBV and associated mean %∆CBV (bar graphs) following AMPH- or saline vehicle- challenge in cortical and subcortical regions of interest. One-way ANOVA followed by post-hoc analyses to test for significant difference in post-AMPH and post-saline responses in HF- vs. LF-fed animals (bar graphs), *p* < 0.05 significant.

As Akt is a downstream target of neuronal insulin receptor activation that regulates surface DAT expression, and, therefore, DA tone, we hypothesized that blunted striatal insulin receptor signaling after 2-weeks on HF diet, as shown by parallel decreases in Akt signaling, would decrease AMPH-evoked neural responses, consistent with decreased surface DAT expression. We therefore examined the effects of HF diet on DAT activity in the brain using AMPH as a probe in an fMRI assay. Specifically, we compared the percent change in CBV over baseline, which reflects neural activity, induced by AMPH in HF- and LF-fed rats. AMPH (3 mg/kg ip) robustly increased CBV across broad cortical and subcortical brain areas in LF-fed rats (Fig 2A), reflecting AMPH-evoked increases in neural activity [5,17–21]. However, in HF-fed rats, AMPH-evoked CBV increases were significantly blunted (Fig 2A), including in the caudate-putamen (CP; 11.5±3.8 vs. 22.1±3.4%, *p* < 0.05) (Fig 2B), motor cortex (M, 41.8±6.1 vs. 21.0±4.7%, *p* < 0.05) (Fig 2C), somatosensory cortex (S, 70.4±12.3 vs. 33.0±5.1%, *p* < 0.01) (Fig 2D), and ventral posterior medial/lateral thalamus (VPM/L, 28.3±6.0 vs. 13.3±4.1%, *p* < 0.05) (Fig 2E). There was no significant CBV response to saline vehicle in either group.

The striatal temporal profiles appeared to differ between HF- and LF-fed rats after minute 20 (Fig 2B). CBV increased over 5–10 minutes to a plateau for both groups, then there was a subsequent monotonic decline in CBV after minute 20 in HF- but not LF-fed rats. However, the slopes of the CP responses were not statistically significant (m_LF_ = +0.00034±0.0013 vs. m_HF_ = –0.0020±0.00054, *p* = 0.063). The decline in CBV observed in NAc in the HF group (Fig 2F) also failed to reach significance (m_LF_ = -0.00020±0.0015 vs. m_HF_ = –0.0029±0.00070, *p* = 0.069).

### High-fat diet degrades integrity of monoaminergic brain networks

**Fig 3:**
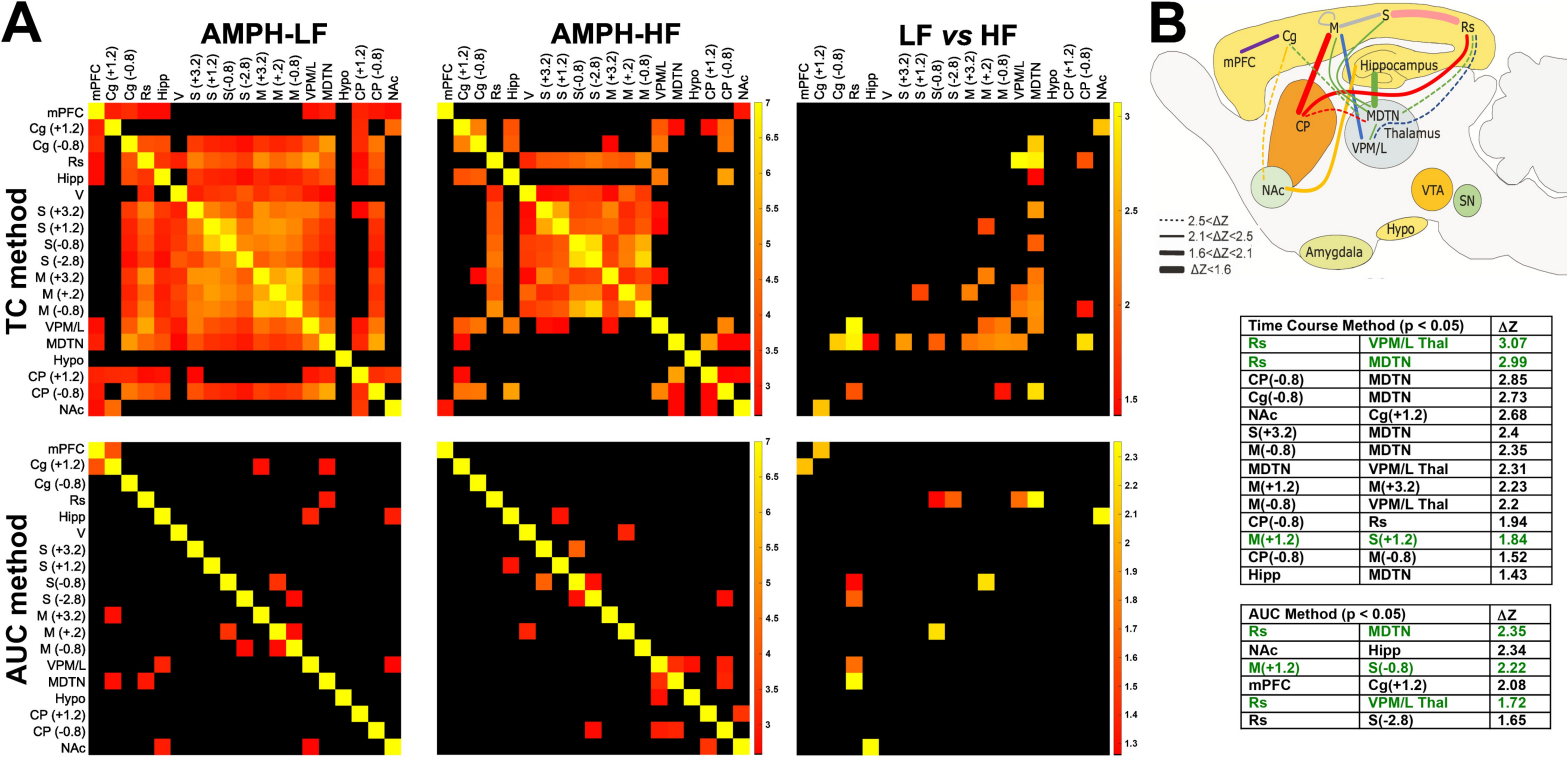
Inter-regional correlations in amphetamine-evoked CBV changes are reduced in HF- vs. LF-fed rats. (A) Correlation matrices identifying brain region pairs where time course profiles (TC; first row) and/or amplitudes (area under curve, AUC; second row) of response to AMPH represent Z-scores that were significantly correlated across animals in LF- and HF-fed rats (*p* < 0.01; first and second columns). For regions present in multiple slices, slice location (mm) relative to Bregma is shown. Significant differences between HF and LF diet groups were found through permutation analysis (LF vs. HF; *p* < 0.05; third column). Abbreviations: mPFC, medial prefrontal cortex; Cg, cingulum; Rs, retrosplenium; Hipp, hippocampus; V, visual cortex; S, somatosensory cortex; M, motor cortex; VPM/L, ventral posterior medial/lateral thalamus; MDTN, mediodorsal thalamic nuclei; Hypo, hypothalamus; CP, caudateputamen; NAc, nucleus accumbens.(B) The size of the effect of HF on inter-regional correlation is depicted by the thickness of the line connecting the region pair in the rat brain schematic. For all region pairs with significant differences between the two groups, the strengths of correlations were weaker in HF vs. LF groups. HF feeding had the strongest effect in region pairs with the largest ΔZ (embedded table) and thickest lines (rat brain schematic). The green font indicates region pairs with significant differences found by both AUC and TC methods.

Analysis of pairwise interregional correlations of CBV responses across animals [20–24] revealed a significant influence of a HF diet on distributed brain circuits for both AUC and TC profile methods. In LF-fed animals (Fig 3A, first column), both AUC and TC profile-based analyses identified broadly similar patterns of inter-regional coupling reflecting striato-cortical (CP-to-sensorimotor (S); NAc-to-cingulum (Cg)), thalamo-cortical (VPM/L-to-S; mediodorsal thalamic nuclei (MDTN)-to-S, and striato-thalamic (CP-to-VPM/L; CP-to-MDTN) circuitry that comprise the mesolimbic/nigrostriatal dopaminergic, fronto-cortical and midbrain-subcortical clusters [20] and AMPH-evoked dopaminergic community structure [22], previously described by Schwarz and colleagues.

Fewer interregional correlations were evident in the HF-fed animals (Fig 3A, second column), with either or both AUC- and TC-based measures of interregional coupling in striato-cortical, striato-thalamic and thalamo-cortical circuits significantly reduced by HF feeding (Fig 3A, third column; Fig 3B and embedded table). HF feeding had the strongest effect, or largest difference between the two groups as indicted by the largest ΔZ score, for the following interregional pairs: retrosplenium (Rs)-to-VPM/L thalamus (ΔZ_TC_ = 3.07; ΔZ_AUC_ = 1.72; Fig 3B), MDTN-to-Rs (ΔZ_TC_ = 2.99; ΔZ_AUC_ = 2.35), CP-to-MDTN (ΔZ_TC_ = 2.85), Cg-to-MDTN (ΔZ_TC_ = 2.73). Significantly weaker correlations for the HF-fed group compared to the LF-fed cohort were also found in dopaminergic regions including: NAc-to-Cg (ΔZ_TC_ = 2.68) and NAc-to-Hipp (ΔZ_AUC_ = 2.34) and, to lesser degrees, CP-to-Rs (ΔZ_TC_ = 1.94) and CP-to-M (ΔZ_TC_ = 1.52).

## Discussion

Our results demonstrate that HF feeding for a relatively brief period significantly impacts the responsiveness of, and functional connections between, distributed brain networks activated by the indirect monoaminergic agonist AMPH. These results are consistent with and extend the mounting evidence implicating the development of impaired insulin signaling and consequent dysregulation of DA signaling in the risk, pathogenesis and/or maintenance of obesogenic feeding behaviors. HF diet perturbed the strength of AMPH-evoked brain activation as measured by amplitude and AUC. The blunted response to AMPH observed in HF-fed animals was most evident in CP and closely resembled the pattern of blunted AMPH-evoked activation that resulted from reduced insulin signaling-dependent surface expression of DAT in insulin-depleted rats [5]. Correlation analyses between ROIs revealed decreased connectivity in mesocorticolimbic dopaminergic brain areas known to subserve reward and appetitive behaviors [25,26]. HF feeding also decreased activity of the insulin-responsive kinase Akt (Fig 1A), a mechanism linked to reductions in striatal DAT surface expression and activity previously demonstrated in a 4-week HF DIO model [8], suggesting that a similar mechanism may contribute to the dysregulated AMPH-evoked brain activation seen in HF-fed animals here. Specifically, depressed insulin signaling may reduce surface DAT expression in striatal and thalamic terminal areas, blunting AMPH-evoked DAT-mediated DA efflux [5, 8] and neural activation (Fig 2). A causal link between impaired insulin signaling and striatal DAT activity is supported by previous studies demonstrating that Akt antagonism blunted DAT activity in normal rats, and that nigral Akt rescue in HF animals restored striatal surface DAT levels, normalized DA clearance, and suppressed hyperphagia [8]. While these preliminary results of the profound effects of brief HF diet on the DA system are interpreted as insulin-mediated, framed in our previous studies on deficits of dopaminergic tone in hypoinsulinemic animals [5,7], they do not exclude the possibility of similar deficits in leptin signaling, consistent with the convergence of the two pathways on Akt [27,28]. We also confirmed that D2R levels (Fig 1B) were lower in HF-fed animals, also consistent with other obesity D2R studies [29, 30] and D2R-dependent mechanisms of insulin [7] and/or leptin signaling [31,32].

To the best of our knowledge, this is the first rodent fMRI report on dopaminergic changes and circuits after a HF diet. Previous DIO studies on the DA system have focused on *ex vivo* striatal slice preparations and/or *in vivo* measurements in a single brain area, and, further, the majority have employed a prolonged HF or other high caloric diet of 4-weeks or longer and consistently reported decreased DA D2R [1,2,4] and reduced DAT function [8,33–36]. Instead of local HSCA used previously [8], here we performed contrast-enhanced CBV fMRI with AMPH challenge to probe DAT function and integrity of DA transmission *in vivo* across multiple brain areas and their circuits. Unlike blood oxygenation level dependent (BOLD) fMRI, which we previously used to probe DAT in our Type 1 diabetes mellitus model [5], the advantages of CBV fMRI include the quantification of a physiological parameter that represents an indirect measure of neural activation and a larger signal window of response. Furthermore, the greater relevance of functional contrast-to-noise ratio over signal-to-noise ratio in CBV fMRI is advantageous for lower field strengths [37]. A multiecho gradient echo sequence was used to measure R2* on a voxel-wise basis because a single TE approach [38] is not ideal for characterizing signal changes in different regions due to the non-uniformity of R2* across the brain. The R2* mapping technique renders more accurate calculations of CBV changes than signal based single TE CBV fMRI, but it is computationally more intensive due to voxel-wise monoexponential fitting at every time point.

Here, for the first time, we show alterations in striatal and extrastriatal areas and their circuits, along with parallel striatal insulin receptor signaling impairment and DA D2R reduction, in a 2-week short-term HF diet model, indicating that these diet-related changes are relatively swift. DIO is associated with changes in DA transmission in brain areas underlying maladaptive behaviors, such as impulsivity, lack of inhibitory control, and habit formation, which may lead to excess food consumption [33, 39–40]. Under scrutiny are the potential mechanisms associated with striatal and prefrontal dysfunction underlying hyperphagia and DIO, including (a) mesolimbic hypoactivity, or reward deficiency [33]; (b) hyperactive responses to food cues [39]; and/or (d) habit formation [40]. Our data indicate that a brief two-week HF diet, compared to LF feeding, affects specific brain areas that underlie such behaviors. For example, decreased striatal D2R availability (Fig 1B) is associated with depressed frontal cortical metabolic rate, both in addiction and obesity [41–43]. Striatal DA is an important modulator of the function of, and coupling between, dorsal attention, frontal-parietal control, and default mode networks [25]. Thus, the impact of HF diet on blunting striatal Akt signaling (Fig 1A) and, consequently, decreased DAT [5] and D2R availability (Fig 1B)–reflected in alterations in striatal DA tone–may have a much broader influence on brain networks central to the regulation of feeding. Indeed, DAT knockdown mice characterized by elevated extracellular DA levels exhibit greater food intake compared to wild-type mice [44,45].

We observed significantly decreased activity in CP in response to AMPH in HF- versus to LF-fed rats, reflecting DAT downregulation. We found no significant difference in the NAc CBV response to AMPH between the HF and LF diet groups, consistent with the results of a previous study showing no change in accumbal DA reuptake or cocaine response in a 2-week HF feeding model [46]. The same study, however, found that after 6 weeks of HF diet, accumbal DAT responses were abnormal [46]. Here, TC-based functional connectivity analysis revealed a significant reduction in NAc-Cg connectivity in HF-fed animals. Future studies should address time-dependence in diet-driven changes in DAT levels and neuroplasticity in the NAc and other regions.

Functional connectivity analyses allowed us to probe correlations between region pairs, or circuits, in responses to AMPH. The interregional correlation patterns in LF-fed animals in the present study recapitulate previous fMRI network studies demonstrating AMPH-associated activation of mesolimbic/nigrostriatal dopaminergic, fronto-cortical, and midbrain/subcortical clusters of brain areas [21,23] that exhibit community structure [24]. Optimal function of these cortico-striatal networks depends crucially on striatal DA tone. The analysis of pairwise circuit correlations revealed additional brain areas significantly impacted by HF diet that were not apparent in single region analysis. HF feeding significantly weakened pair-wise correlations between subcortical and cortical (Rs, Cg, and to a lesser degree M) structures, including NAc-to-Cg. The reduced functional coupling of striatal signaling with specific cortical and subcortical AMPH-evoked responses, combined with the broad vulnerability of a fronto-cortical network receiving mesolimbic/nigrostriatal inputs observed in HF-fed animals, lends support to this idea.

## Conclusion

Our findings demonstrate that a brief HF diet rapidly dysregulates dopaminergic brain networks underlying key components of feeding-related behaviors, including reward, motivation, and cognitive control. The parallel blunting of central insulin signaling suggests HF-associated impairment in central insulin receptor signaling and consequent compromise of dopaminergic function as a potential pathogenic pathway to obesogenic feeding behaviors. Future studies can address rescue of insulin signaling-mediated DA dysregulation in HF-fed animals and behavioral phenotypes of short-term high-fat diets.

## Abbreviations

AMPH: amphetamine
AUC: area under the curve
CBV: cerebral blood volume
Cg: cingulum
CP: caudate putamen
D2R: D2 receptor
DA: dopamine
DAT: dopamine transporter
DIO: diet-induced obesity
fMRI: functional magnetic resonance imaging
FOV: field of view
GSK3ɑ: glycogen synthase kinase 3 alpha
HF: high-fat
HSCA: high-speed chronoamperometry
ip: intraperitoneal
iv: intravenous
LF: low-fat
M: motor
MDTN: mediodorsal thalamic nuclei
NAc: nucleus accumbens
NE: norepinephrine
NET: norepinephrine transporter
PET: positron emission tomography
R2*: transverse relaxation rate
ROI: region of interest
Rs: retrosplenium
S: somatosensory
TC: time course
TR: repetition time
VPM/L: ventral posterior medial/lateral
